# The association between back pain and trunk posture of workers in a special school for the severe handicaps

**DOI:** 10.1186/1471-2474-10-43

**Published:** 2009-04-29

**Authors:** Kelvin CH Wong, Raymond YW Lee, Simon S Yeung

**Affiliations:** 1 Department of Rehabilitation Sciences, The Hong Kong Polytechnic University, Hong Kong; 2 School of Human and Life Sciences, Roehampton University, UK

## Abstract

**Background:**

The present study aims to determine the time spent in different static trunk postures during a typical working day of workers in a special school for the severe handicaps.

**Methods:**

Eighteen workers with low back pain (LBP) and fifteen asymptomatic workers were recruited. A cross-sectional design was employed to study the time spent in different static trunk postures which was recorded by a biaxial accelerometer attached to the T_12 _level of the back of the subjects.

**Results:**

The results of ANCOVA revealed that subjects with LBP spent significantly longer percentage of time in static trunk posture when compared to normal (p < 0.05). It was also shown that they spent significantly longer time in trunk flexion for more than 10° (p < 0.0125).

**Conclusion:**

An innovative method has been developed for continuous tracking of spinal posture, and this has potential for widespread applications in the workplace. The findings of the present investigation suggest that teachers in special schools are at increased risk of getting LBP. In order to minimise such risk, frequent postural change and awareness of work posture are recommended.

## Background

Low back pain (LBP) is a prevalent and disabling work-related musculoskeletal complaint encountered by a variety of industries. Epidemiological studies reveal that LBP is related to awkward postures, including trunk flexion with or without rotation [1-4], combined trunk flexion and manual lifting [5,6], frequent trunk bending [7] and prolonged static trunk flexion [8,9]. It has been shown that prolonged static trunk flexion may subject the spine to reduced activity of multifidus [10], provoke flexion relaxation phenomenon of the thoracic erector spinae resulting in the creep response of the lumbar spinal tissues [11-13], reduce the oxygenation of lumbar extensors due to the constant isometric contraction [14], and increase the intradiscal pressure [15].

Questionnaires and interviews, observation and direct measurements are commonly used techniques to document work postures at the workplaces. Bussmann et al. [16] stated that each of these approaches had its practical as well as methodological limitations. For instance, the use of questionnaires may give rise to low reliability and validity result [17] and the subjects may overestimate their postural exposure [18]. Observation method can only provide a rough idea of the time spent in certain postures [17] and it is only suitable for monitoring occupations that normally confine in small working area. Although the inclinometer has shown to be reliable in measuring lumbar spine range of motion [19,20], the temporal characteristics of the spinal movement cannot be documented. Advancement in technology has enabled the development of miniature accelerometers that can be suitably mounted onto the human body to record both static and dynamic human movement. Indeed, accelerometer has been used in measuring the trunk postures and movements in ergonomic research [21,22].

Working at floor level with stooped posture clearly constitutes a health risk for the musculoskeletal system of radish harvesting workers [23] and preschool workers [24]. To the authors' knowledge, no previous study has examined the risk factors of LBP for teaching staff in special schools although LBP is commonly reported in this workforce. Most students in the special school suffered from multiple contractures and deformities and are dependent on the teaching staff for carrying out the basic activities of daily living. Thus, the teaching staff members are required to spend much time in performing manual transfer and mat activities for the students in a stooped posture. These tasks may pose high risk to the development of cumulative trauma to the back. Based on the policy of the school, each teaching staff is expected to share the similar physical work load as students are evenly distributed into different classes based on their age and body weight. Thus, the work posture that they adopted during a typical working day might contribute to the development of LBP at the workplace.

The purpose of the present study was to develop an objective accelerometer-based method to record the static trunk posture in a typical working day of workers in a special school, and to compare the time spent in different static trunk postures between subjects with and without back pain.

## Methods

### Study design

A cross-sectional design was conducted to record the duration of time spent in different static trunk postures among the teaching staff of a special school for severe handicaps.

### Subjects

All the staff members involved in the classroom routine of a special school for the severe handicaps were invited to join the present study. Subjects were included if they are full time staff and had more than one year of working experience in this school or other special school with similar setting. Subjects were excluded from the present study if they suffered from back pain or injury that was not work-related. Fifty subjects met this selection criteria and 44 of them volunteered to join the study. To ensure the subjects are having the similar exposure to manual lifting at work, the staff members were asked about the total number of lifts that they need to perform each week. The teaching staff members with similar lifting number were included in the current study. A total of 33 subjects (21 teachers, 3 teaching assistants and 9 health care professionals) were recruited for this study. They were divided into two groups: 18 subjects who suffered from bilateral LBP for at least 1–7 days in previous 12 months and severity greater than 1 in the pain scale (0–5), and 15 subjects who did not have back pain in the previous 12 months.

The demographic data of the subjects are summarized in Table 1. T-test revealed no significant differences in mean age, height, body weight, and number of lifts performed each day between the two groups. Nonetheless, there was significant greater proportion of female subjects, and marginal shorter years of working experience in the LBP group.

**Table 1:** Descriptive statistics of subjects' demographic characteristics

Characteristic	Subjects with LBP (n = 18)	Asymptomatic subjects (n = 15)	p-value
Mean age (SD)	33.9 (8.2)	38.9 (8.0)	0.085
Sex (%)			
Male	4 (22.2%)	9 (60%)	
Female	14 (77.8%)	6 (40%)	0.03
Mean height (SD)	161.4 cm (7.9)	163.4 cm (6.9)	0.448
Mean body weight (SD)	53.5 kg (9.5)	58.5 kg (10.2)	0.157
Mean years of working experience	8.5 (4.8)	12.0 (5.6)	0.061
Average number of lifts per shift	16.5(2.9)	15.3(2.3)	0.20
Professions	Teacher = 11 Health care professional = 5 Teaching assistant = 2	Teacher = 10 Health care professional = 4 Teaching assistant = 1	

### Ambulatory postural monitoring system

**Figure 1:**
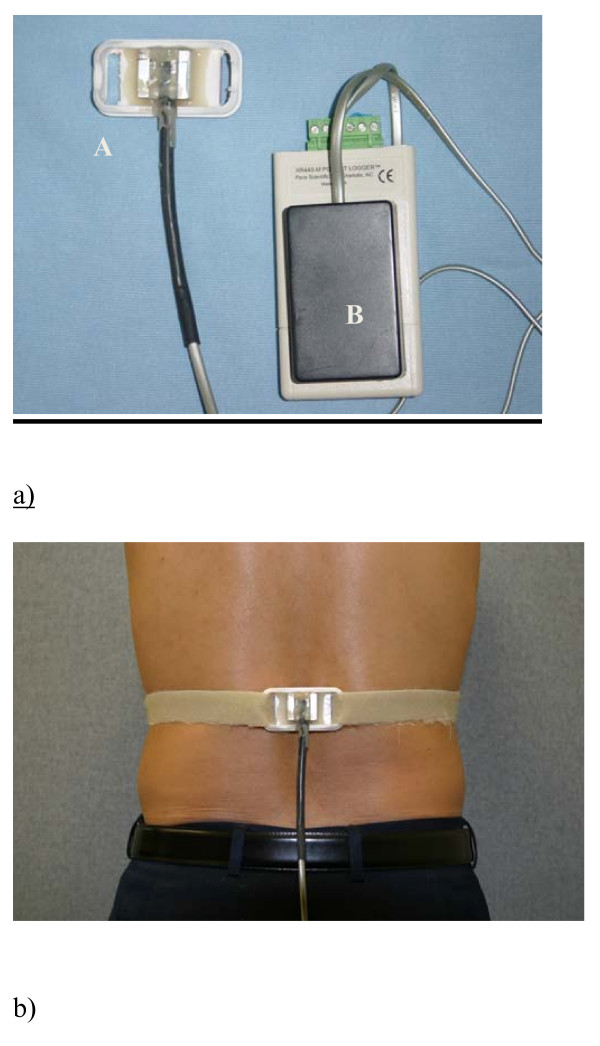
**Ambulatory postural monitoring device**. a) the accelerometer (A) and the data logger (B). b) Strapping for secure attachment of the accelerometer to the back.

A bi-axial accelerometer (ADXL202JE, Analog Devices Inc. USA) and a data logger (Pace XR440, Pace Scientific Inc. USA) were attached to a plastic plate which was housed in a small aluminum cube. A Velcro soft strap was threaded through the plate so that when it encircled the trunk, its tension could provide extra stability for accelerometer attachment and to minimize the movement between the accelerometer and the underlying skin (Figure 1). The accelerometer was aligned with the spinous processes so that it was not tilted sideways. Since the accelerometer was tilted with respect to the horizontal, the signals in upright standing were taken and this angle of the accelerometer measurement was defined as zero flexion. All measurements were made with respect to this inclination.

### Calibration of the accelerometer and pilot testing

The accelerometer was calibrated and pilot tested before it was applied to record the static back postures of the workers during a typical work day. The accelerometer was mounted on one arm of a standard goniometer, and was calibrated for static angle measurement by acquiring its signals when positioned at known inclinations from ± 180° with 15° increment. As pure plane of sagittal flexion and extension is not common for human movement, the same procedure of calibration was repeated with the accelerometer side tilted for 15° and 30°. The averaged difference between the angles of the standard goniometer and the calculated angles from the signals of the accelerometer was small (0.34° ± 0.25°).

In static condition, the sensor detects the vertical inclination relative to gravity [25,26]. The vertical inclination (θ) of the accelerometer is given by



where acc_y _is the acceleration in the y-direction and acc_x _the acceleration in the x-direction. As constant acceleration is virtually impossible during normal dynamic activities, conditions will be assumed to be dynamic if the accelerometer detects a time-varying signal and static if the signal is relatively constant. Based on the above assumption, an experimentally determined threshold has to be applied to differentiate the static and dynamic nature of the activities. Activity is defined to be dynamic if the fluctuation of signal is greater than the threshold and static if it is well lower than the threshold [16,26,27]. For this reason, we conducted a pilot test aimed at determining the threshold in defining the static activities from the raw signals of the accelerometer. Three subjects were randomly selected. The accelerometer was attached to the T_12 _level of the back of the subjects by double-sided adhesive tape. The sampling rate of the data logger was set to be 5 Hz. The subject was then asked to stand upright statically for 1 minute. The signals obtained were regarded as "static standing". After that, eight static postures were performed for 30 seconds (standing with 30°, 60° trunk flexion, sitting with 0°, 30°, 60° trunk flexion, kneeling with 0°, 30°, 60° trunk flexion). The raw signals obtained were low-pass filtered with a cutoff frequency of 0.2 Hz by the second-order Butterworth filter [28]. Thirty seconds of the resultant signals of the x and y axes from the known period of "static standing" were extracted. The mean absolute range of the signals was used as the threshold for defining the static posture of that subject.

An algorithm was proposed to define the activity as dynamic in nature if all the absolute differences between the adjacent signals within one-second data were greater than the threshold. The threshold was adjusted until the proposed algorithm could correctly detect more than 95% of time period of the eight static conditions for the three subjects.

The algorithm was then validated by the 3SPACE Fastrak system (Polhemus, Colchester, VT, USA) in discriminating the static and dynamic conditions. The procedures are essentially the same as the setup for the calibration procedures. In brief, the accelerometer and the Fastrak sensor were mounted on one arm of a standard goniometer. The goniometer was either kept in static at various positions or rotated forward and backward for 6 trials. The input signals of both the accelerometer and Fastrak were synchronized and AD converted to a computer.

The algorithm used for the determination of the onset of dynamic conditions was compared to the signals from the Fastrak sensor. The absolute differences between the accelerometer and Fastrak in detecting the time of onset and stop of the six dynamic conditions were calculated and averaged. It was found that both the accelerometer and Fastrak system could consistently detect the various static and dynamic conditions and the averaged difference between the accelerometer and Fastrak in detecting the time of onset and stop was small (0.69 ± 0.57 s). The accelerometer was considered to be sufficiently accurate for detecting static and dynamic postures.

**Figure 2:**
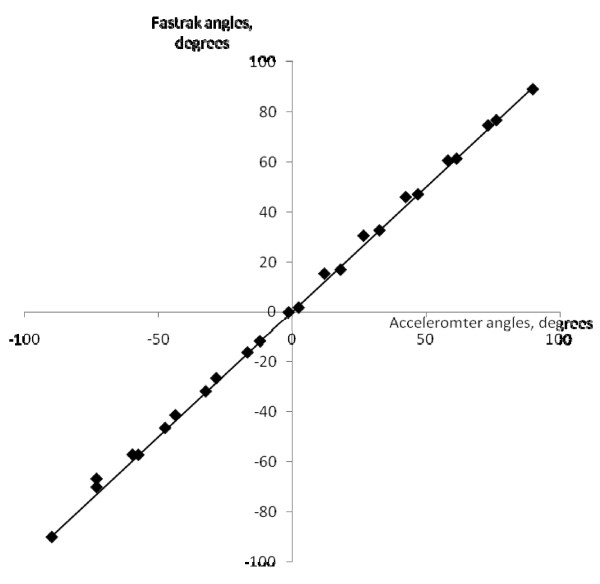
**Plot of fastrak angles against angles derived from the accelerometers with the best straight line fit**.

The Fastrak was also used to validate the angles derived from the accelerometer. Figure 2 shows a plot of the two sets of inclination angles during various static positions. The data was fitted with a best straight line (slope = 0.99, intercept = 0.2°), with a correlation coefficient of 0.99. This indicated that there was very little offset error in the accelerometer measurement, and there was very strong agreement between the two data sets.

### In-field trunk posture measurement

The accelerometer was attached at T_12 _level by using the double-sided adhesive tape. Before the attachment, the skin was cleaned by cotton wool soaked with 75% alcohol. The position of the accelerometer was then marked on the back by using the black ball pen. The position of the accelerometer was further secured by asking the subjects to fasten the strapping and apply the soft corset clothing over the trunk himself. With the tension of the strapping and the soft corset clothing, the stability of accelerometer on the back would be enhanced and excessive movement of the accelerometer on the back could be prevented. The subject were also asked to check if there was any discomfort or hindering of trunk movement by the strapping and soft corset so that he could not perform the trunk movement "naturally".

Prior to the experiment, subjects were asked to stand still for 1 minute. Signals recorded were used to determine the threshold of static posture for that subject. It is important to note that the threshold should be individually determined because different people may adopt different body sway during their static activities. The subject was then asked to resume his work in normal pace. Postural data was collected during the morning (3 hours) and afternoon (3 hours) period of the subject at a sampling rate of 5 Hz. By the end of the working day, the subject was asked to stand straight statically again for 1 minute and this postural data was used to compare with that of the initial standing position to ensure there was no difference between two data set. Furthermore, the accelerometers were employed as inclinometers when the subject was detected to have adopted a static posture. The angle of trunk inclination was calculated from the signals of the accelerometers as described above.

The accelerometer signals were checked in upright standing after the experiment. The accelerometer would detect a proportion of acceleration due to gravity (g) according to its inclination with respect to gravity. If the accelerometers had been moved due to skin or soft tissue deformation or insecure attachment, the accelerometer signal in upright standing would be different after the experiment. So checking the signal in upright standing would allow us to determine if the accelerometer had moved away due to the error.

### Data analysis

The percentage of time spent in static trunk posture was calculated by dividing the time spent in static trunk posture by the total time of postural data collection of the whole working day. Moreover, the percentage of time spent in static trunk posture was further categorized into four groups of trunk flexion, namely (1) θ ≤ 10°, (2) 10°<θ ≤ 30°, (3) 30°<θ ≤ 60°, (4) θ > 60°, where θ is the inclination angle.

Statistical analyses were conducted using the Statistical Package for the Social Science (Version 16 SPSS Inc. Illinois USA). As there was a greater proportion of female subjects (p = 0.03) and shorter years of working experience (p = 0.061) in the LBP group, gender and working experience were employed as covariates in the analysis. Two way analysis of covariance (ANCOVA) was used to analyze the effects of group (back pain vs asymptomatic) and posture (the four posture categories i.e. θ ≤ 10°, 10°<θ ≤ 30°, 30°<θ ≤ 60°, θ > 60°) on the percentage of time spent in these postures. The interaction between the variables group and posture was examined, and post-hoc analysis was performed if the independent variables were found to be significant (p < 0.05).

## Results

### In-field trunk posture measurement

**Figure 3:**
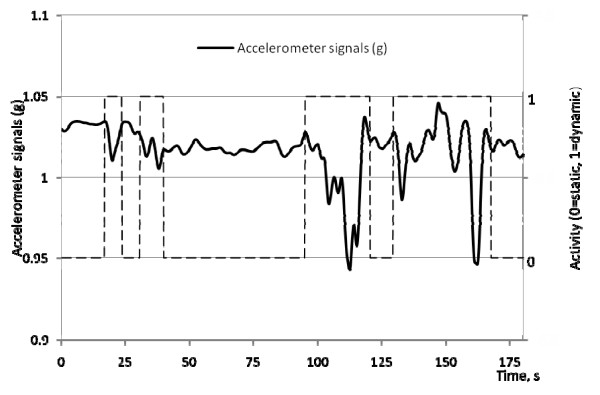
**Plot of the resultant accelerometer signals against time**. The period when the signals exceed the threshold (shown as dotted line with a value of 1) was defined as dynamic activities.

Figure 3 shows the raw data of the resultant accelerometer signals against time in one of the subjects. It clearly demonstrates that using the algorithm described above, the accelerometer was able to effectively discriminate static and dynamic conditions. The period when signals exceeded the threshold (shown as a dotted line with a value of 1) was defined as dynamic activities, whereas static conditions was denoted by the dotted line with a value of 0.

The angles of trunk inclination of the known period of static standing before and after the work for all of the subjects were compared by intra-class correlation coefficient (ICC). High correlation was shown between the two trials of static standing [ICC(1,1) = 0.92]. Moreover, the accelerometer did not move away from the skin markings after the whole day work for all of the subjects. Thus, the accelerometer was fixed properly on the back of the subjects during the whole day work without any unwanted movement between the skin and the accelerometer.

**Figure 4:**
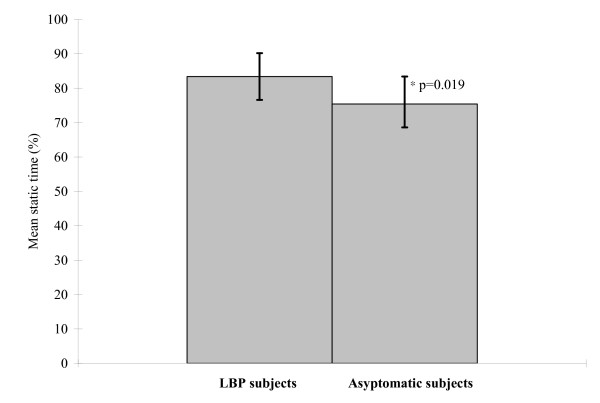
**Percentage of time spent in static back posture for the LBP and asymptomatic subjects**.

The percentages of time spent in static trunk posture for the two groups of subjects are summarized in Figure 4. ANCOVA revealed a significant difference in percentage of time spent in static trunk posture between the 2 groups and interaction between the group and posture (p < 0.05). Subjects with low back pain spent longer time in static trunk posture than the group without LBP. Post hoc analysis revealed significant differences between the 2 groups in percentage of time spent in all four trunk posture categories (p < 0.05) (Table 2). The results are summarized in Table 2. LBP subjects spent significantly less time in posture with flexion of less 10° and more time in the other three posture categories with flexion of more than 10° (p < 0.05).

**Table 2:** Duration and percentage of time in static back posture for LBP subjects and asymptomatic subjects in four trunk flexion categories

	Subjects with LBP (n = 18)		Asymptomatic subjects (n = 15)		p-value
≤ 10° flexion	3.41 ± 0.83 hr	56.9 ± 13.7%	4.61 ± 0.93 hr	76.8 ± 15.6%	0.002
11°–30° flexion	1.86 ± 0.59 hr	31.0 ± 9.9%	1.09 ± 0.74 hr	18.2 ± 12.4%	0.012
31°–60° flexion	0.69 ± 0.34 hr	10.2 ± 5.7%	0.25 ± 0.18 hr	4.2 ± 3.0%	0.002
≥ 61° flexion	0.11 ± 0.12 hr	1.9 ± 2.0%	0.03 ± 0.02 hr	0.5 ± 0.4%	0.012

Total	6 hr	100%	6 hr	100%	

## Discussion

### Tracking spinal posture in the workplace

It is generally accepted that cumulative spinal loading is one of the causative factors contributed to LBP [29,30]. Thus, it is important to accurately record the cumulative effects of static postures at the workplace such that appropriate intervention strategies can be introduced. This study employs a new, innovative method to track static posture using accelerometers. The results of the current study indicated that the accelerometer-based method is accurate and feasible at the workplace. It can record the temporal characteristics and the cumulative effects of the static spinal movement at the workplace over an extended period of time. This is superior to the questionnaires or observation methods commonly used in the field which only provide a subjective estimate of time spent in work postures. The accelerometers employed in this study are small, light and inexpensive, and are ideal for tracking spinal posture in the workplace. Future study should explore how these sensors can be used in other applications and work environments.

### The association between LBP and static trunk postures at work

In the present study, staff with LBP spent significantly longer percentage of time in static trunk posture with more trunk flexion when compared to staff without LBP. It thus appears that an increase in risk of back pain may be associated with long period of sustained stooped posture. Indeed, similar results had been shown in other studies. For instance, Christensen et al. [31] applied both questionnaires and observation method for addressing the reason of occurrence of LBP among the workers in the Danish wood and furniture industry. Their results indicated that manual material handling and prolonged static trunk flexion for more than 10 seconds were the main reasons for the workers to suffer from LBP. Similarly, Hoogendoorn et al. [9] investigated the relationships between trunk posture and lifting at work and the occurrence of LBP by video-taping the work postures of the blue- and white-collar workers. They showed that the workers had higher risk of getting LBP when they worked with the trunk in a minimum of 60° flexion for more than 5% of their working time (RR = 1.5). In a three year prospective cohort study, Hoogendoorn et al. [4] found an increase in risk (OR = 1.4) for the workers to get low back pain when there was an increase in time of exposure to heavy load and flexed trunk posture. In a ten year longitudinal study, Marras et al. [3] showed that the reduction of the maximal sagittal trunk flexion of the workers by the introduction of lift tables could significantly decrease the low back disorder incidence rate. Clearly, a sustained posture of stooping is a major factor associated with the occurrence of LBP.

As the staff in the special school is required to adopt a flexed trunk posture on the ground level for performing mat activities for the students, the external flexion moment of the trunk is inevitably counteracted by the activation of the lumbar extensors and the passive structures of the spine. Such muscle contraction may further increase when there is external loading at the trunk when workers are required to support the severely handicapped students during various functional training. Constant contraction of the erector spinae during static lumbar flexion, even at the level of 2% of MVC would compromise the capillary blood flow to the erector spinae, and predispose the muscle to fatigue and injuries [14,32]. Callaghan and McGill [32] pointed out that as a result of fatigue, LBP may result from stress accumulation of the passive structures that stimulates the nociceptors responses [12] and the lactate accumulation over the erector spinae due to the effect of deoxygenation of the muscles [14]. Prolonged lumbar flexion will also lead to creep and other viscoelastic responses [11] so that spinal tissues are subjected to higher strains, and the risks of injuries may be increased.

The results of the present investigation and the previous studies support the conclusion that teaching members of this special school are at increased risk of getting low back pain when they spend prolonged period of time in static trunk flexion during work. Frequent postural change and awareness of trunk posture during mat activities are highly recommended for relieving the stress of passive and active structures of the low back resulted from constant loading of the static trunk posture. Rest activities such as standing up from stooping and walking for a short distance are suggested to promote the cyclic muscular contraction and relaxation that facilitate the nourishment of spinal tissues and provide periodic rest to the muscles [32].

It should be noted the present study only examined the static trunk postures of the teaching staff. The effects of external loading onto the spine were not examined. However, giving the fact that the subjects performed similar manual handling activities at work, the effects of external load should be common to both the symptomatic and asymptomatic group. It is also impossible to use accelerometers to record dynamic trunk orientation and this will require other inertial sensors such as gyroscopes. Further study is suggested to include the measurement of dynamic trunk motions of the subjects so as to examine the role of dynamic movements in back pain. Moreover, a self-reported work-task diary is suggested to record the work task of subjects so that the tasks with high risks of injuries can be specifically identified and a more specific ergonomic intervention can be suggested.

## Conclusion

An innovative method has been developed for tracking spinal posture. This study demonstrated that the accelerometer-based method is highly sensitive in differentiating the static and dynamic nature of activities. It is highly accurate in recording static spinal posture as well as the time spent in these postures. The sensors are also small in size, light in weight and inexpensive, and will be the ideal tool for continuous monitoring of trunk posture in ergonomic and clinical assessments.

It was shown that teaching staff with LBP spent significantly longer percentage of time in static trunk flexion (more than 10°) than those without LBP. It is concluded that back pain is associated with prolonged periods of time in static trunk flexion during work. Frequent postural change and awareness of trunk posture are recommended for relieving muscle fatigue and tissue strains due to constant loading of the spine.

## Competing interests

The authors declare that they have no competing interests.

## Authors' contributions

KCHW, RYWL, and SSY participated in conceiving the study design, data analyses and interpretation of the data. KCHW contributed substantially in the data collection, and drafted the manuscript. RYWL and SSY have been involved in critically revising the manuscript for important intellectual input. All authors read and approved the final manuscript.

## Pre-publication history

The pre-publication history for this paper can be accessed here:


